# Effect of Wettability and Uniform Distribution of Reinforcement Particle on Mechanical Property (Tensile) in Aluminum Metal Matrix Composite—A Review

**DOI:** 10.3390/nano11092230

**Published:** 2021-08-29

**Authors:** Johny James, A. Raja Annamalai, A. Muthuchamy, Chun-Ping Jen

**Affiliations:** 1 Department of Mechanical Engineering, Sri Venkateswara College of Engineering & Technology, Chittoor 517127, India; johnyjames2002@yahoo.com; 2 Centre for Innovative Manufacturing Research, Vellore Institute of Technology, Vellore 632014, India; raja.annamalai@vit.ac.in; 3 Department of Metallurgical and Materials Engineering, National Institute of Technology, Tiruchirappalli 620015, Tamil Nadu, India; muthuchamy@nitt.edu; 4 School of Dentistry, College of Dental Medicine, Kaohsiung Medical University, Kaohsiung 80708, Taiwan; 5 Department of Mechanical Engineering and Advanced Institute of Manufacturing for High-Tech Innovations, National Chung Cheng University, Chia-Yi 62102, Taiwan

**Keywords:** aluminum, composite, microstructure, mechanical properties, porosity

## Abstract

There is a massive demand for low-weight high strength materials in automotive, space aerospace, and even structural industries in this present engineering world. These industries attract composites only because of their high strength, resistance to wear, and low weight. Among these composites, metal matrix composite finds wide applications due to its elevated properties, excellent resistance property, corrosion resistance, etc. The reinforcements exist in particles, fiber, and whiskers. Among the three, particles play an important role because of their availability and wettability with the metal matrix. Additionally, among the various metal matrices such as aluminum, magnesium, copper, titanium, etc., aluminum plays a vital role among metal matrices because of its cost, availability in abundance, and castability. Stir casting is the most inexpensive and straightforward composite fabrication technique among the prevailing techniques. Even though so many factors contribute to the elevated property of composites, metal matrix, and reinforcement phase, uniform distribution and wettability are essential factors among all the other factors. This review aims to develop a composite with elevated property in a cost-effective manner. Cost includes metal matrix, reinforcement, and processing technique. Various works have been tabulated to achieve the above objective, and analysis was carried out on tensile strength concerning microstructure. This review paper explores the challenges in composite fabrication and finds a solution to overcome them.

## 1. Introduction

Technological innovation, the higher speed with less fuel consumption, the need for elevated property, and costs are the motivations for inventing newer materials. For the aerospace and automotive industry, weight reduction is an essential requirement that paves the way for new reinforcements and metal matrix to develop metal matrix composite. Indeed, composites are not new but have existed for many centuries. Old Testament records the use of straw in the fabrication of brick before 5500 years. Notwithstanding, the bulk production of composites was before three decades only. Composites are a combination of mainly two main constituents, i.e., metal matrix phase and reinforcement phase. Judicial selection of the reinforcement type, particle size, and metal matrix phase and fabrication technique results in the elevated property [1].

### 1.1. Category of Composites

Composites are generally categorized into three main categories based on the matrix substance. They are called metal matrix composite (MMC), polymer matrix composite (PMC), and ceramic matrix composite (CMC), respectively [2]. MMC gives more elevated ductile properties than CMCs and better environmental stability than PMCs. Additionally, MMCs offer good thermal conductivity (from 220 to 580 W·m^−1^·K^−1^), wear resistance (0.01025), wear rate (g·m^−1^), erosion, and shear strength [3,4,5,6,7,8,9,10].

### 1.2. Matrix Materials in MMCs

Since the day work on metal matrix composites commenced, aluminum and its alloys played a vital role as matrix materials due to the increase in demand for high-strength, lightweight components. Similarly, magnesium and titanium alloys are also employed as metal matrix material, but both have their demerits because magnesium quickly reacts with the atmosphere, so processing is complicated; as for titanium, it is highly reactive and forms inter-metallics with many reinforcement materials [11,12].

### 1.3. Reinforcements

Suitable reinforcing material needs to be chosen to fit a given material. The standard form of reinforcements is particles, whiskers, and fibers (continuous or discontinuous). The particles develop the property of the composite by dispersion strengthening and by hindering the movement of dislocations. Additionally, when reinforcing materials are added as particles, they impart isotropic properties. The commonly employed reinforcements are SiC, Al_2_O_3_, TiB_2_, and B_4_C, etc.

### 1.4. Applications of MMCs

Based on the statistics, most aircraft components have been replaced by composites, and a considerable amount are MMCs. Additionally, many automotive engine manufacturers already replaced forged steel with MMCs. Piston, piston ring, connecting rod, brake rotor, cylinder liner bearings, bushings, etc., are some of the components made by MMCs due to their wear resistance, high strength, specific stiffness, and fatigue strength [13,14].

### 1.5. Production of MMCs

Various processing techniques are employed in the fabrication of MMCs. They are powder metallurgy, diffusion bonding, spray co-deposition, and casting routes. The casting route is one of the most common and economical from the above fabrication techniques even though it has limitations like porosity and agglomeration defects. Stir casting is commonly acknowledged as a proven technique, presently adopted commercially as well. In addition, stir casting permits a conventional metal processing technique to be integrated or replaced; this leads to cost reduction [15]. Furthermore, this liquid casting method is a low cost compared to the other entire composite fabrication method [16] and permits huge dimensional components.

Skibo, M reported that the price of producing composites using a casting route is almost 1/3 to 1/2 of other existing routes, but for colossal size production, it may even fall to 1/10 of the cost by other methods. Even though it has few limitations, they can be overcome by using stir casing integrated with a squeeze casing unit [17,18].

### 1.6. Various Metal Matrix and Reinforcement Phases of Advanced Composites Using Different Techniques

H. Abdizadeh processed composite using A356 aluminum alloy and ZrO_2_ by stir casting method. The vol.% of reinforcement varies from 5 to 15 percent, and the temperatures were 750, 850, and 950 °C, respectively. It was reported that aluminum alloy reinforced with ZrO_2_ particles enhanced UTS and hardness compared to A356 aluminum alloy, and its maximum values were 232 MPa and 70 BHN, respectively. For 15 vol.% of ZrO_2_ composition, the highest UTS, and hardness values were obtained [19].

Baghchesara, M. A produced composite using A356 aluminum alloy and ZrO_2_ by stir casting method. Various samples of 5, 10, and 15 volume percent of ZrO_2_ in different casting temperatures of 750, 850, and 950 °C were prepared. The maximum tensile strength was recorded in the sample having 15 vol.% ZrO_2_ prepared at 750 °C, which shows an enhancement of 60% compared to the aluminium-356 parent alloy. Additionally, it has been concluded that the composite fracture was severely brittle by increasing ZrO_2_ particle quantity and casting temperature [20].

M. Hajizamani fabricated composite using A356 and ZrO_2_ by stir casting method. In this work, nanoparticles of ZrO_2_ and Al_2_O_3_ were constituted to produce composites with a composition of 0.5–2 wt.% of the reinforcement. This work records that by raising the reinforcement, density reduced while yield, ultimate tensile strength (UTS), and compressive strength improved. The ductility of the composite specimens was low due to high porosity and the formation of voids. Additionally, hardness improved at one weight percent of Al_2_O_3_ and 10 weight percent of ZrO_2_. However, for the hardness of the specimen at 1.5–2 weight percent of Al_2_O_3_ and 10 weight percent ZrO_2_ again the hardness value came down [21].

G.Karthikeyan selected aluminum LM25 as the parent material, reinforced with 0–15% of zirconium oxide prepared by stir casting route. Wear and tensile specimens were made per ASTM G99 and ASTM B-557-M-94 standard. A surface roughness test was done on wear specimens. The test shows that a rise in ZrO_2_ particles percentage promotes surface roughness value [22].

Using the stir casting method, S. Prajval prepared a metal matrix composite by combining aluminum A356 and titanium dioxide (TiO2) with various mica percentages (1%, 2%, 3%, 4%, and 5%). It has been concluded that the UTS value is highly influenced by the percentage of mica and TiO_2_ present in the composite. Additionally, the UTS value is noteworthily influenced by the process of heat treatment and aging method. The hardness of the specimens increases when the reinforcement in the composite specimen increases [23].

T. Rajmohan studied the property of hybrid A356 metal matrix composite reinforced with mica and SiC particles. Micrographs were investigated with the help of SEM. EDX was used to study the material composition. The results showed that better hardness was obtained for the composition of 10 wt.% of SiC and 3 wt.% of mica. The rise in weight percent of mica enhances the wear-resistant property of the composite [24].

R. Raj processed 6061Al-B_4_C, the composite containing different wt.% of B_4_C using advanced stir casting method with bottom pouring set-up. The dispersion of particles of B_4_C in the aluminum matrix, interfacial characteristics, and microstructural features was qualitatively studied using an optical microscope and field emission scanning electron microscope (FESEM). Microstructural characterization revealed that the distribution of B_4_C in the metal matrix phase was comparatively uniform, and at some locations, small-scale agglomeration and clustering of particles were observed. Particle size distribution has been studied for the quantitative description of agglomeration of B_4_C particles, revealing small-scale agglomeration of particles. Homogeneity and randomness of B_4_C particulates across the matrix phase have been calculated by the quadrant method. The results show a random spatial distribution of particles with small-scale clustering [25].

B. P Beyrami fabricated composite using A356 and ZrO_2_ nanoparticles. Samples of composites were made at different percentages of ZrO_2_ (1.5, 2.5, and 5 vol.%). The casting temperatures were selected as 800–950 °C. Micrographs of composite samples were studied using scanning electron microscopy (SEM) and energy dispersive X-ray spectroscopy (EDS). Mechanical properties such as compressive yield strength, toughness, and hardness were calculated. The experimental results depict that mechanical properties like compressive yield strength and hardness are noticeably picked up by adding ZrO_2_ particles. The highest values were for samples containing 2.5 vol.% of particles fabricated at 850 °C [26].

Two sets of cast composite specimens were prepared by stir casting fixing the 10% of fly ash and varying (5% and 10%) zirconia by weight fraction. S. Malhotra et al. reported that the optimum casting conditions of the composite fabrication were attained with 10 wt.% ZrO_2_ and 10 wt.% fly ash. There is a considerable increase in tensile, elongation, and hardness value [27]. M. Ramachandra synthesized composite using Al 6061 and ZrO_2_ to study corrosion behavior by the stir casting process. This work reports that the corrosion rate of the parent material is higher than the cast composite, and the best property is achieved in 7.5 wt.% ZrO_2_ specimen [28].

D. M. Patoliya prepared composite using Al 6061 and ZrO_2_ by the stir casting process. Four specimens were prepared to vary wt.% from 0 to 7.5, keeping all other parameters the same. It is also reported that tensile strength, hardness, and impact strength have been promoted parallel to the rise in weight fraction of zirconium oxide particles in the Al 6061 matrix, but elongation decreased with increase in wt percent of ZrO_2_ in the Al 6061 matrix [29].

P. R Thyla fabricated composite using Al 6061, Gr, SiC, and ZrO_2_ by the stir casting process. Five different samples were prepared to study the corrosion behavior. It has been reported that the corrosion rate decreased due to the presence of ceramic particles in the matrix material. Specifically, 9% wt percent sample records a very minimum corrosion rate [30].

The above works of various researchers show the development of composites using aluminum alloy and various reinforcements with different particle sizes and compositions. It proves the successful development of composite. One of the main objectives of composite is to achieve high strength. Table 1 gives a detailed report of the metal matrix, reinforcement, and the tensile strength achieved.

**Figure 1:**
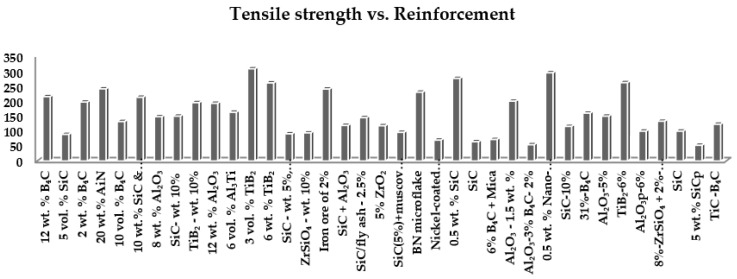
Graph of several reinforcements versus tensile strength [31,32,33,34,35,36,37,38,39,40,41,42,43,44,45,46,47,48,49,50,51,52,53,54,55,56,57,58,59,60,61,62,63,64,65,66].

From Figure 1, the highest strength reported is by M. K Akbari et al. using metal matrix A356 reinforced with 3 vol.% TiB_2_ that results in 308 MPa and K. Amouri using metal matrix A356 reinforced 0.5 wt.% Nano-SiC that results in 295 MPa. The lowest strength reported is by V. Singh using aluminum alloy reinforced with 5% (weight) SiCp that results in 52.8 MPa, and B.V Ramnath using aluminum alloy reinforced with Al_2_O_3_—3% B_4_C—2% and achieved a tensile value of 54.6 MPa. The above experimental work listed in Table 1 shows a vast deviation in tensile strength. Various factors can cause variation in tensile strength. This work analyzes the most influential factors, i.e., wettability, uniform distribution, cluster formation, etc., in detail [31,32,33,34,35,36,37,38,39,40,41,42,43,44,45,46,47,48,49,50,51,52,53,54,55,56,57,58,59,60,61,62,63,64,65,66].

### 1.7. Analysis on Micrographs

A microstructure exhibits the quality of bond in between the reinforcement phase and metal matrix phase. Additionally, the formation of the third phase is identified with the help of a micrograph which influences tensile strength. Holding time or temperature enables the formation of the third phase, which contributes to tensile strength. Since wettability and uniform distribution enhances tensile property, it is worth having an in-depth analysis of micrographs.

**Figure 2:**
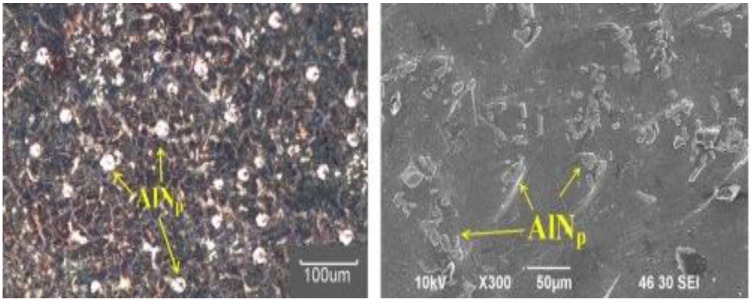
Micrographs of AA6061-AIN composite.

Kumar, B. A reported that clustered aluminum nitride reinforcing particles are distributed consistently in the aluminum alloy 6061 matrices, as shown in Figure 2. The proper interface is observed between the reinforcement particle and the AA6061 matrix. Mechanical property is improved due to AIN particles. The tensile strength reported is 193–241 MPa.

**Figure 3:**
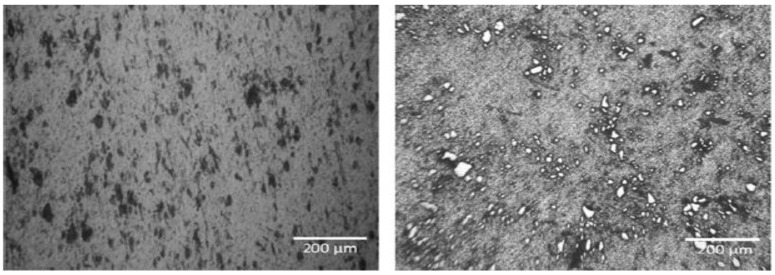

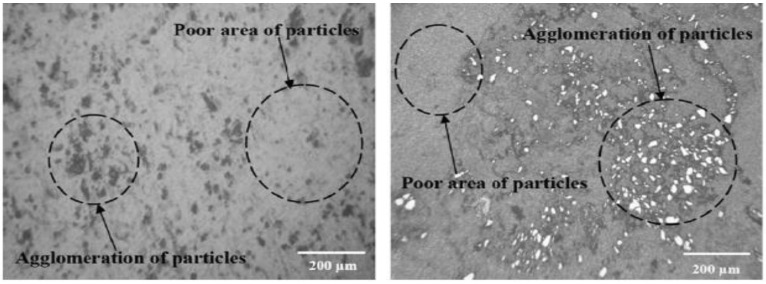
The micrographs of Al–TiC–B_4_C composite.

Mazaheri Y reported that the oxide film stops the aluminum melt from attaining intimate contact with the reinforcement, and the breakdown of the oxide layer at high temperatures is essential to attain wettability. This can be accomplished by adding flux Na_3_Al F_6_ along with reinforcement particles to aluminum melt simultaneously. Still, particle agglomeration and uneven allocation of particles in the matrix are shown in Figure 3. Habitually, the particles created agglomerates; this can only be dispersed by high rpm stirring with limited time and proper melt temperature. The melt’s enhanced wetting and complete covering of reinforcement particles by the melt can be obtained by heating B_4_C particles and adding flux along with TiC particulates. The tensile strength reported in this work is 123–132 MPa.

**Figure 4:**
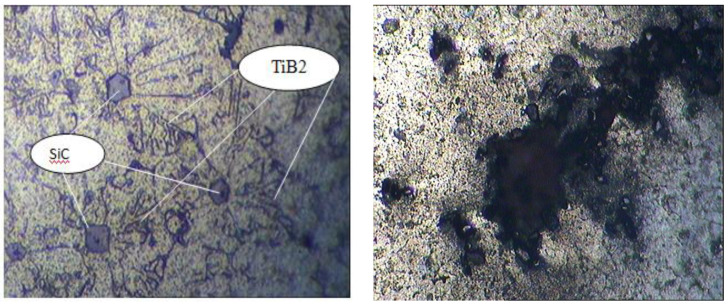
Micrographs of AA6061-SiC-TiB_2_ composite.

James, S. J reported that TiB_2_ ceramic particles are particular for superior strength; the test proves low ultimate tensile strength. This is due to the surplus cluster formation, which results in pore formation. Casting parameter components such as stirring blade speed, the temperature at the time of holding, impeller position into the melt, and the dimension of the impeller are the essential parameters that must be considered during the aluminum composite fabrication. These factors influence tensile property. Additionally, rise in weight percentage of particle reinforcement up to 15% is one of the causes for cluster formation, as shown in Figure 4. The tensile strength reported is 150.1 MPa.

**Figure 5:**
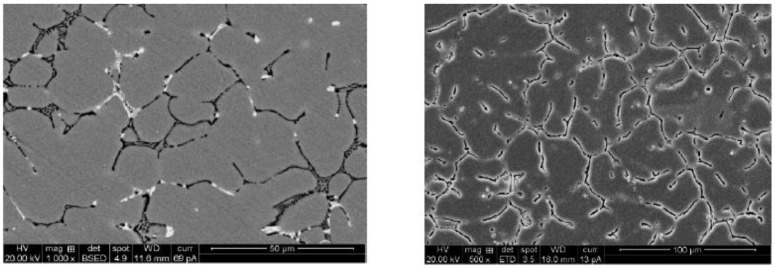
Micrographs of 6061Al-Al_2_O_3_ composite.

Bharath V reported that for each composite specimen, the reinforcement phase is preheated to 200 °C and then added little by little in three steps into the vortex of aluminum 6061 alloy melt to increase wetting behavior and to achieve uniform dispersion. Microstructures of cast composites were studied by taking samples from the middle part of the casting to ensure particle dispersion. Tensile property and hardness of the developed composite specimens are measured. Micrographs of the developed composite specimen prove even dispersion and a little measure of grain refinement in the developed composites, as shown in Figure 5. The recorded tensile value is 193 MPa.

**Figure 6:**
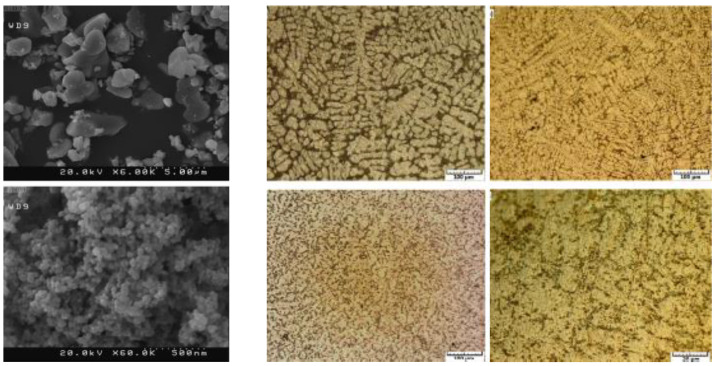
The micrographs of the A356-TiB_2_ composite.

Akbari, M. K reported that the microstructural study proves that the nanoparticles are to some extent pressed by Al dendrites to the uttermost freezing areas, as shown in Figure 6. Nanocomposites substantially show dissimilar tensile properties when matched up with that of the microparticle reinforced specimens. When matching up with the parent material, noteworthy enhancements in tensile strength value are recorded. Micro composites show almost lower and unvarying toughness values that match up with that of the parent material. Due to a rise in porosity content, the tensile strength of the composite substantially diminished with a rise in processing temperature. The tensile value reported is 308 MPa.

A list of factors that influence the tensile value is mentioned and discussed in detail.

#### 1.7.1. Selection of Metal Matrix Phase

Thermodynamically stable dispersoids are crucial for the metal matrix composites which are to be used in the high-temperature application. This can be performed only by an alloy dispersoid arrangement in which interfacial energies, solid-state diffusivity, and elemental solubility are reduced, minimizing coarsening and interfacial reactions. Magnesium and aluminum alloys are mainly metal matrices due to their low-density value and high thermal conductivity properties. Composite with low matrix alloying additions affects an impressive combination of ductility, toughness property, and strength. A small amount of alloying elements is utilized in wrought alloys as grain refiners are not essential in metal matrix composites having discontinuous phases [67].

Grain refiners lead to the creation of coarse intermetallic compounds at the time of consolidation, which reduces the ductility property of composite during tension. Many of the metals and alloys are good choices for matrices. Nevertheless, in practice, the preference for low-temperature applications is low. Light metals are receptive due to their low density, etc. Ti, Al, and Mg are the accepted matrix materials present in the application, mainly functional for defense and aircraft applications. The high strength of metal matrix materials mainly depends upon high modulus reinforcing materials. The strength-to-weight ratios of these composites are higher than in their alloy form. The preference for reinforcing materials becomes more stunted with the rise in the melting temperature of metal matrix materials. The matrix phase is a considerable material wherein ceramic reinforcing particles are dispersed and are continuous. Especially in structural purposes, the matrix phase is generally a lightweight metal such as Al, Mg, and Ti, supporting reinforcement [68,69].

#### 1.7.2. Preferred Property of Matrix Phase

The preferred property of the matrix phase in a composite structure is excellent flow characteristics, minimum moisture absorption, low shrinkage, low thermal expansion, sound strength, elongation and modulus, high strength at elevated temperature, good chemical resistance, processable and dimensional stability, etc.

#### 1.7.3. Factors Are Taken into Account during the Selection of Matrix Phase

During the selection of matrix phase, these are the factors that need to be taken into account. They are the matrix, and the reinforcement phase must be well-suited. Hence, if a high-strength fiber is chosen as the reinforcement phase, there is no meaning in choosing a low strength matrix phase, which fails to transmit stresses proficiently to the reinforcement phase. The matrix phase should meet the service environment such as ultra-violet, humidity, temperature, chemical, atmospheric conditions, abrasion, etc. The matrix should be compatible with the preferred casting technique. The final cast composite must be less expensive [70].

#### 1.7.4. Selection of Reinforcement Phase

Reinforcement should give strength to the composite, which is the meaning and purpose of reinforcement. Reinforcement can also supply certain additional features of conduction, corrosive resistance and rigidity, etc. Reinforcement that decorates the matrix phase should be stiffer, more robust, and talented in converting failure to the produced composite. Or in other words, the ductility must be minimized to perform as brittle as possible. The option of reinforcing materials is further stunted with the rise in the melting temperature of matrix phases [71].

#### 1.7.5. Percentage Composition of Reinforcement

The percentage of the composition of reinforcement with the metal matrix can be in volume or weight. It can be varied from 1 to 40%. The increased percentage will end up with cluster formation or agglomeration. This can be prudently decided from previous related work. This work has reported 99 percent density and even distribution of reinforcement for cast composite having 15% volume fraction [72]. An increase in the composition of reinforcement particles leads to cluster formation or agglomeration.

### 1.8. Size of Reinforcement

Results of various research reveals that increases in particle size result in a reduction of the strength of the composite. The strength obtained by varying particle size by 0−20, 40–80, and 80–100 µm is 295, 195, and 250 MPa, respectively. A decrease in particle size significantly impacts the hardening rate, which developed dislocation tangles around the particle phase. The distance between the particles (λ) reduces smaller particle sizes at a steady quantity of reinforcement particles. Particle size reduction increases barriers against the grain boundary movement; this leads to a reduction in grain boundary movement [73,74,75].

### 1.9. Shape of Reinforcement

Following the theory of strain gradient of Wang-Chen, an organized investigation on the effect of particle size in metal matrix phase composite reinforced with different particle phases was performed. A detailed investigation was carried out on various composite factors, such as matrix material strain hardening exponent, particle aspect ratio, particle size, the volume fraction of particles, etc. Spheroidal and cylindrical-shaped particles were taken into account to find the strength-dependence concerning shape [76]. Shape decides the bonding between reinforcement and matrix phase.

### 1.10. Wettability

**Figure 7:**
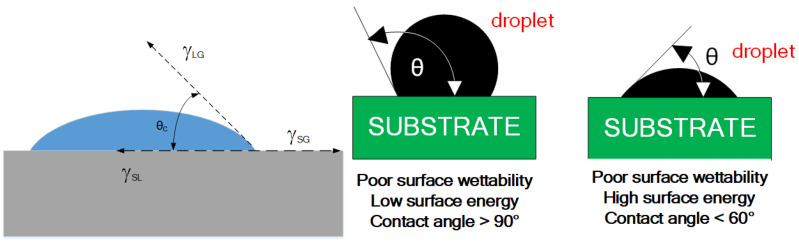
Wettability in terms of contact angle.

Wettability defines the amount to which a liquid will spread around a solid surface. When the liquid (matrix) flows over the reinforcement phase covering the rough surface entirely and removes all air, it is termed good wettability. Close contact between a solid and liquid may be achieved through this, provided the liquid does not possess viscosity. From this, it can be understood that wetting must occur first to have any adhesion. A high value of free energy can prevent the wetting of a liquid on a surface on the liquid surface. The balance of forces is derived from contact angle (q) or wet, as shown in Figure 7, by a Young equation [77].

### 1.11. Surface Tension and Surface Chemistry

**Figure 8:**
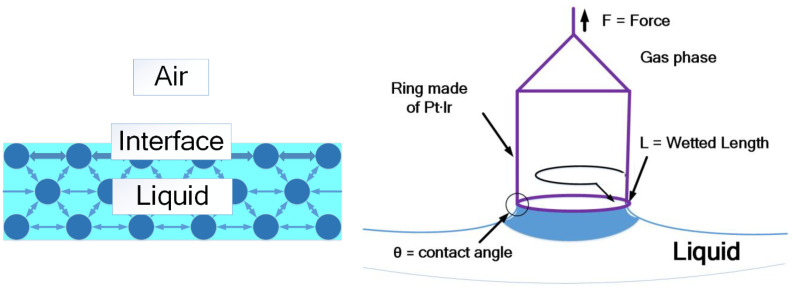
Surface tension.

Measurement of cohesive energy available at any interface is termed surface tension. The equation to find surface tension is stated in Equation (1). Figure 8 describes the surface tension.

ϒ = F (force exercised parallel to the surface of the liquid in Newton)(1)

L (line of action of force in meters)

### 1.12. Interface Bonding

Mechanical interlocking may lead to bonds of reasonable stability, depending on the roughness of the surface. Mechanical interlocking may be particularly beneficial to shear strength development, whereas it is not very stable concerning regular forces. Electrostatic bonding interaction between charges at the molecular level—act only at minimal distances [78]. Chemical bonding forms a covalent bond between matrix and reinforcement (ionic 600–1100 KJ covalent 60–700 KJ/mol).

### 1.13. Prolonged Contact Time between the Matrix and Reinforcement Phase

An interfacial chemical reaction develops undesired third phases. Formation of undesired third phases can be avoided by choosing a chemically attractive or compatible combination of materials. Additionally, elevated temperatures reduce the contact time between material phases.

One of the significant conditions in selecting constituents for composite concoction is that both matrix and reinforcement phases should be chemically inert and non-reactive. It is difficult to synthesize composites having ingredients with divergent linear expansion characteristics. When it comes to interface, the contact area between reinforcement and matrix materials is termed as interface. Particularly in some situations, the contact region is a distinct added phase. Some composites offer interphases when different surface ingredients interact with each other. Choice of synthesis method depends upon the matrix property and matrix effect upon the property of reinforcements.

### 1.14. Particle Matrix Interface Energy

The author M. Huang investigates the effect of stress concentration concerning particle shape and size. Stress concentration will be present inside the particle and at the interface. It is stated that normal interfacial SCF to a certain point and interfacial energy is minimum [79]. This work concludes that the interface energy affects the quality of matrix and reinforcement.

### 1.15. Selection of Processing Route

Processing metal matrix composite using stir casting will be the most straightforward and less expensive processing method [80,81].

### 1.16. Stir Casting Parameters

Stirring speed is an essential parameter because this enhances wettability between matrix and reinforcement phase and supports even circulation of reinforcement phase across the matrix. After a wide range of experiments, the optimal stirring blade speed has been concluded to be from 400 to 500 rpm. Likewise, the stirring temperature needs to be slightly higher than the metal matrix’s melting point to hold the melt at the necessary viscosity level. Stirring or holding time should not be more than 5–10 min [82,83].

Kumar, M. P conducted simulation-based studies to understand the consequence of stirring blade speed on particle dispersion across the matrix phase. Silicon carbide nano reinforcement was used as a reinforcing material and copper as a metal. The simulations were performed by changing the stirrer speed from 200 to 400 rpm while fixing other factors constant such as blade angle 60° and viscosity 4.4 mPa·s. Particles were distributed evenly at 400 rpm for the above parameters [84].

### 1.17. Uniform Dispersion

Achievement of uniform distribution is the main challenge in composite concoction with the help of stir casting. Uniform distribution is one of the highest influential factors on mechanical property. Judicial optimization of process parameters and selection of phases with proper composition can be achieved [85].

### 1.18. Inclusion of Slag

Usage of the bottom pouring technique is one of the methods to overcome this slag inclusion. Additionally, slag should be removed before pouring.

### 1.19. The Chemical Affinity between the Metal Matrix and Reinforcement Phase

A chemical reaction between the matrix and reinforcement phases should coincide with various other factors. If an acceptable interface bond between reinforcing materials and matrix is not obtained, this represents a failure in manufacturing. This can be due to the disparity between the physical properties of the metal matrix phase and reinforcement phase and fabrication methods. Additionally, different thermal expansion coefficients of the matrix and reinforcement can produce residual stresses in the composite during the fabrication process. If the thermal expansion coefficients are dissimilar, thermal cycling can induce stresses in the metal matrix composite. Most of the systems, including boron–magnesium, boron–aluminum, SiC–aluminum, etc., were noticed. Surface energy difference can cause poor wetting of reinforcing ceramic particles by the molten liquid metal, which leads to a weak structure in the composites.

Another problem is the formation of eutectic compounds due to the reaction between the molten metal and the particle surface. Carbon aluminum is an example of third phase formation and some other challenges. In a composite fabrication system, poor wettability is a significant challenge. Though above 1000 °C wettability increases, above 500 °C chemical reaction between aluminum and carbon will be induced, resulting in brittle aluminum carbide. Application of coatings to the reinforcement particles and by constituting alloying element controls reactions between the reinforcing particles and matrix phase since the reaction process is by diffusion.

For instance, the reaction between molten aluminum and carbon fibers can be rectified by co-deposition of boron and titanium on the carbon fiber just before composite preparation. The painting of surface coatings on the reinforcement particles improves wettability. For example, boron fibers can be coated to develop wetting, coating of B_4_C on boron fibers controls reaction with aluminum and titanium matrices, and application of silicon carbide coating on boron fibers minimizes reaction in between fiber and matrix. Modifying the matrix composition will enhance wetting between the matrix and reinforcement and chemical bonding. Wetting can be enhanced by allowing selected impurities to combine or react with the fiber or particle surface, which can be wetted or spread over by the matrix material. A small quantity of about (2–3%) of lithium into the aluminum matrix to support bond or adhesion with aluminum oxide fibers is a proven example of this method and applies to particles. Lithium reacts with alumina and forms lithium aluminates; this is easily wet by the aluminum. To enhance the wetting of carbon fibers, low melting point metals like lead, indium, and thallium can be added to aluminum [15,86,87,88,89,90,91,92,93,94,95].

### 1.20. Density Difference, Viscosity, Design, and Position of Blade and Gating System

Density differences between matrix alloys melt, and reinforcement particles result in uneven distribution of particles in the matrix phase. Additionally, various designs of blades contribute to a uniform distribution. Stirring speed can decide the transfer of reinforcement into the melt, and it can hold the reinforcement particles in a condition of suspension. The position of the stirrer from the bottom of the furnace affects uniform distribution. The stirrer should be placed so that 35% of liquid should be under the stirrer and 65% liquid should be above the stirrer. Additionally, fabrication temperature influences the melt viscosity. The uniform particle distribution varies concerning viscosity change. Structure formation during solidification is influenced by pouring temperature, which decides the metal flow rate. A proper gating system needs to be arranged to have a free melt flow [96,97].

### 1.21. Casting Environment

Stirring blade speed and stirring time reflect in the microstructure formation during casting. The even allocation of reinforcement particles across the aluminum matrix relies primarily on stir casting parameters and rate of cooling concerning the type of casting method employed. A quick-quench compo caster is recommended while processing high-temperature materials.

### 1.22. Rearrangement of the Reinforcement Particles during Solidification

Theoretical models predict the pre-settling of nanoparticles at slower rates. Various experiments prove that the melt matrix temperature has an impact on the settling behavior during casting. Conducted experiments depict the inverse relationship between the Vol fraction of reinforcing materials and temperature near melting point with settling rate. The viscosity of both metal and reinforcing material mix reduces as the melt mixture temperature increases. Due to this viscosity reduction, the reinforcement particle phases try to stay down concerning volume or weight percent composition. During solidification settling, time must be minimized [98,99,100,101].

### 1.23. Accuracy of Experiments

There are so many factors that decide the reliability of tensile values. This includes the trustworthiness of certified purity of reinforcement and matrix, accuracy and precision of the testing machine, number of samples tested, and its calculations—the specimen size, whether macro or micro, decides tensile results. The specimen can be made from the best part of the cast sample. The genuineness of the values also reported a critical concern [102].

## 2. Conclusions

Tensile property is one of the vital properties among the other mechanical properties for a metal matrix composite. It can be achieved by judicial selection of metal matrix, i.e., type of aluminum. Reinforcement selection too plays a vital role in the strength of processed composite. Besides these, the casting techniques, i.e., stir casting or stir casting, coupled with squeeze casting, are influential factors. The stir casting parameters need to be judicially selected and optimized to obtain uniform distribution and excellent wettability. This factor decides the tensile property of the processed composite. The formation of the third phase also has considerable influence on the tensile property of processed metal matrix composite.

## Figures and Tables

**Table 1:** Various tensile strength values of developed composites.

S.L. No	Author	Metal Matrix	% and Reinforcement	Tensile Strength in MPa	Reference
1	Kalaiselvan (2011)	AA6061	12 wt.% B_4_C	215	[31]
2	Amirkhanlou and Niroumand (2011)	A356	5 vol.% SiC	89	[32]
3	Alizadeh (2011)	Al	2 wt.% B_4_C	197	[33]
4	Kumar (2012)	AA6061	20 wt.% AlN	241	[34]
5	Mazaheri (2013)	Pure Al	10 vol.% B_4_C	132	[35]
6	Selvam (2013)	AA6061	10 wt.% SiC and 7.5 wt.% flyash	213	[36]
7	Kumar (2013)	A359	8 wt.% Al_2_O_3_	148	[37]
8	James, S. J. (2014)	Al 6061	SiC—10 wt.%	150.1	[38]
9	James, S. J. (2014)	Al 6061	TiB_2_—10 wt.%	195	[39]
10	Bharath (2014)	AA6061	12 wt.% Al_2_O_3_	193	[40]
11	Yang (2015)	A356	6 vol.% Al_3_Ti	163	[41]
12	Akbari (2015)	A356	3 vol.%TiB_2_	308	[42]
13	Niranjan (2015)	A356	6 wt.% TiB_2_	261	[43]
14	James, S (2017)	Al 6061	SiC—5 wt.%, Al_2_O_3_—3 wt.%, TiB_2_—2 wt.%	91	[44]
15	JohnyJames, S (2017)	Al 6061	ZrSiO_4_—10 wt.%	94	[45]
16	Ansar Kareem (2021)	AA 6061	Iron ore of 2%	240.5	[46]
17	Vipin Kumar Sharma (2019)	AA 6061	SiC + Al_2_O_3_	119	[47]
18	S Narendranath (2020)	AA6061	SiC/fly ash—2.5%	145	[48]
19	S. Roseline (2018)	Al6061	5% ZrO_2_	118	[49]
20	Sharma (2021)	Al–Mg–Si–T6	SiC (5%) + muscovite (2%)	96.08	[50]
21	Konopatsky (2021)	AlSi10 Mg	BN microflake	230	[51]
22	Sha, Jian-jun (2021)	Al Alloy	Nickel-coated carbon fiber	70	[52]
23	Rao (2021)	Al7075	0.5 wt.% SiC	276	[53]
24	Kumar (2021)	Al–SiC	SiC	64.55	[54]
25	Velavan (2021)	Al	6% B4C + Mica	72	[55]
26	Ezatpour (2014)	Al6061	Al_2_O_3_–1.5 wt.%	200	[56]
27	Ramnath (2014)	Al Alloy	Al_2_O_3_–3% B_4_C–2%	54.6	[57]
28	Amouri, K (2016)	A356	0.5 wt.% Nano-SiC	295	[58]
29	El-Sabbagh (2013)	6061/F500	SiC–10%	115.66	[59]
30	Yu, L. I., et al. (2016)	AA1100	31%–B4C	160	[60]
31	Kandpal (2017)	AA 6061	Al_2_O_3_-5%	150	[61]
32	Sumankant (2017)	A356	TiB_2_–6%	261.84	[62]
33	Alaneme (2013)	AA 6063	Al_2_O_3_p–6%	100	[63]
34	Rino (2013)	AA 6064	8%–ZrSiO_4_ + 2%–Al_2_O_3_	132.98	[64]
35	Kumar, G. V (2010)	Al6061	SiC	100	[65]
36	Singh, V (2004)	AA 6061	5 wt.% SiCp	52.8	[66]
37	Mazaheri, Y (2013)	Al	TiC–B4C	123	[35]

## Data Availability

The data will be shared upon request from the authors.
